# Phylogeography and Ecological Niche Shape the Cichlid Fish Gut Microbiota in Central American and African Lakes

**DOI:** 10.3389/fmicb.2019.02372

**Published:** 2019-10-15

**Authors:** Laura Baldo, Joan Lluís Riera, Walter Salzburger, Marta Barluenga

**Affiliations:** ^1^Department of Evolutionary Biology, Ecology and Environmental Sciences, University of Barcelona, Barcelona, Spain; ^2^Institute for Research on Biodiversity (IRBio), University of Barcelona, Barcelona, Spain; ^3^Zoological Institute, University of Basel, Basel, Switzerland; ^4^Department of Biodiversity and Evolutionary Biology, Museo Nacional de Ciencias Naturales, CSIC, Madrid, Spain

**Keywords:** allopatry, sympatry, Mida cichlid, continent, Illumina 16S rRNA, *Amphilophus* spp.

## Abstract

Cichlid fishes, with their repeated colonization of lakes and subsequent radiations at different scales of phylogenetic and ecological diversification, offer an excellent model system to understand the factors shaping the host-gut microbiota association in nature. Here, we characterized the gut microbiota of the *Amphilophus* species complex from Central America (known as the Midas cichlid complex), encompassing 158 wild specimens (13 species) collected from seven Nicaraguan lakes, and combined these data with previously published data from two African lakes (spanning 29 species). Our aim was to comprehensively explore trends in microbiota variation and persistence along the large spatial and temporal scales of cichlid diversification (from the oldest radiation in L. Tanganyika, 9–12 My old, to young ones in Nicaraguan crater lakes, <0.5 My old), in allopatry and sympatry (within and across lakes), and across the range of dietary niches (from highly specialized to generalist feeders). Despite their extraordinary diversity, cichlids shared a remarkably conserved microbial taxonomic profile, which argues for a primary role of the host genetics in the assembly and maintenance of these microbial communities. Within this partly constrained microbiota profile, geographic isolation (continent and lake) represented the first level of discrimination. For the Midas cichlid, a partial congruency was found between host microbiota and genetic distances, suggesting that microbial communities have partly diversified along their cichlid phylogeographic history of crater lake colonization. In sympatry (within lakes), the young and poorly ecologically diversified cichlid assemblages of Central American lakes display largely unresolved gut microbiotas (in terms of both alpha and beta diversities), whereas the phylogenetically and ecologically diverse species found in African lakes showed greater microbial interspecific diversity. This pattern largely points to the level of habitat segregation, trophic niche overlap, and reproductive barriers as major modulators of the gut microbiota connectivity among sympatric species.

## Introduction

Over the past decade, our understanding of the diversity and functional role of host-associated microbial communities has dramatically changed, and with it the way we interpret organismal biology. This paradigm shift urges researchers to revise and complement studies in both animals and plants, from the molecular field to life history traits, in virtue of the microbes they live with (Gilbert et al., 2015; Shapira, 2016; Macke et al., 2017; Smith et al., 2017). Microbial effects can result on dramatic changes on the host fitness (Hurst, 2017; Rosshart et al., 2017; Gould et al., 2018), supporting the important role of microbes in promoting or enhancing animal adaptation, and potentially facilitating diversification (Shropshire and Bordenstein, 2016; Pennisi, 2017).

In particular, studies of gut microbiota have increased in the past few years, revealing – among other results – strong correlations between gut community structure and host diet, geography and phylogeny, or a combination of such factors (Ley et al., 2008; Delsuc et al., 2014; Sanders et al., 2015; Smith et al., 2015; Brooks et al., 2016; Moeller et al., 2016). Vertebrate adaptive radiations, i.e., the rapid formation of novel species from a common ancestor as a consequence of the adaptation to new ecological niches (Gavrilets and Losos, 2009), represent a powerful natural system to explore the eco-evolutionary dynamics of this symbiotic association at relatively short temporal scales and along a set of ecologically diverse, yet closely related species. Few vertebrate adaptive radiations have been explored so far, including the iconic Galapagos finches (Michel et al., 2018), the *Anolis* lizards from Puerto Rico (Ren et al., 2016), the *Podarcis* lizards from the Balearic Islands (Baldo et al., 2018), and cichlid fishes from African lakes (Baldo et al., 2015, 2017).

Cichlids are a well-known model system to study ecological diversification (Fryer and Iles, 1972; Seehausen et al., 2008; Salzburger, 2009, 2018), offering multiple examples of adaptive radiations triggered by adaptation to distinct ecological niches, and resulting in extraordinary morphological, behavioral and ecological parallelisms (e.g., Muschick et al., 2012). A large fraction of their taxonomic diversity is presently found in the African Great Lakes Tanganyika (approximately 250 recognized species), Malawi and Victoria (>700 species each) (Salzburger et al., 2005; Salzburger, 2009, 2018), while less species-rich cichlid assemblages are known from rivers and many smaller lakes in the tropics and sub-tropics. In Central America, cichlids are present in several lakes, which contain much younger and less diverse fish assemblages (ca. 20 species, with the most recent lake radiation dating back to <10,000 years) (Barluenga and Meyer, 2004, 2010; Kautt et al., 2016b, 2018).

The great range of cichlid phylogenetic and ecological diversity along with their geographic distribution, all in the context of a single fish family (Cichlidae) (Brawand et al., 2014; Salzburger, 2018), make them a particularly interesting system to study the dynamics of the gut microbial communities across both spatial and temporal scales of variation.

We recently characterized the gut microbiota of African cichlids from Lake Tanganyika and crater Lake Barombi Mbo (in Cameroon), showing that the structure and diversity of these microbial communities largely correlated with both cichlid geography (lake) and major dietary niches (suggesting a key role in the transition between carnivory and herbivory), with important parallelisms in microbial community changes seen across lakes (Baldo et al., 2017).

**FIGURE 1:**
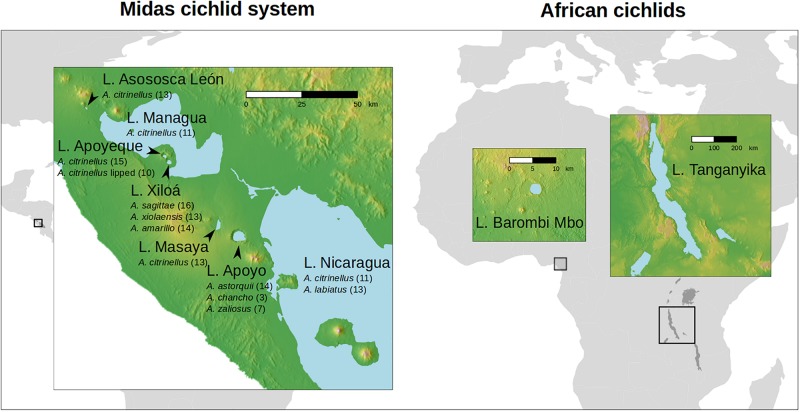
Map of the sampled lakes for the cichlid gut microbiota (seven Central American and two African lakes). Total samples (*N* = 274 specimens) encompassed 42 species. For Midas cichlids, sampled species (*N* = 13) and specimens (*N* = 158) are given below the lake name (number of individuals per species is shown in parentheses, see Supplementary Table S1 for metadata). For the two African lakes, Barombi Mbo and Tanganyika, data were obtained from Baldo et al. (2017) (specimens, *N* = 116, for details on species and diet see Supplementary Table S1 of this previous study).

In the present study we explored for the first time the gut microbiota of the Midas cichlid species complex (*Amphilophus* spp.) from Central America. This cichlid species complex inhabits the two large Nicaraguan lakes (L. Nicaragua and L. Managua) and several smaller lakes of volcanic origin that have formed along the Pacific Ring of Fire (Figure 1). These radiations are younger and conspicuously smaller compared to some of the African cichlid assemblages (including L. Tanganyika and Barombi Mbo), accounting for a unique genus, *Amphilopus*, which has diversified into approximately 20 species/morphotypes within the past 0.5–1 My (Kautt et al., 2018). The large Nicaraguan lakes, Nicaragua and Managua (0.5 My, intermittently connected through the River Tipitapa; see Figure 1), currently host the species *A. citrinellus*, the most common and widespread type, and *A. labiatus*, restricted to rocky areas and characterized by hypertrophied lips and elongated heads. *A. citrinellus* from the large lakes independently colonized the crater lakes formed in the collapsed calderas of inactive volcanoes during the past ca. 50,000 years (Barluenga and Meyer, 2004, 2010; Kautt et al., 2016b). Within the remarkably deep crater lakes (mean depth varies between 17.2 and 142 m; Barluenga and Meyer, 2010), cichlids have diverged in full sympatry along the benthic-limnetic axis, evolving in parallel similar eco-morphotypes in different lakes and giving rise to the characteristic benthic and limnetic forms (Elmer et al., 2010; Kautt et al., 2016a). This ecological divergence, promoted by both the distinctive geological features of the crater lakes (deeper and with clearer water compared to the shallower and more eutrophicated large lakes), marked founder effects and recurring bottlenecks following episodes of mass extinction, has led to the local appearance of endemic species, with some of the most derived phenotypes seen in the well-studied crater lakes Xiloá and Apoyo (Barluenga and Meyer, 2010; Kautt et al., 2016a). According to their relatively recent morphological and ecological diversification, all Midas cichlid species are largely omnivorous (MB, unpublished data) and do not show the extreme trophic specialization seen in several African species (i.e., no true carnivores or herbivores are found).

The newly obtained microbiota data from Central America (158 specimens from 13 species found in seven lakes) was here combined with previously published data from African cichlids (116 specimens from 29 species found in two lakes) (Baldo et al., 2017) to explore the structuring and changes of the gut microbiota as a function of phylogeography (between continents and among lakes), trophic ecology (highly specialized diet *versus* omnivory) and reproductive barriers (good species *versus* incipient species/morphotypes).

Some of the major questions we aimed to address were the following:

Do cichlids share a conserved microbial profile, regardless of their collective geographic, phylogenetic and ecological variables?

Can the gut microbiota diversity alone predict the Midas cichlid omnivorous diet, as previously shown for African carnivores and herbivores?

Did the Midas cichlid microbiota diversify in parallel with the lake colonization history and subsequent host speciation within lakes?

## Materials and Methods

### Samples, DNA Extractions and Sequencing

A total of 189 fish specimens were collected between November and December 2016 in seven Nicaraguan lakes, including the two large lakes, Nicaragua and Managua, and five crater lakes present in the western part, right on the Pacific Ring of Fire (Figure 1, see Supplementary Table S1 for sample metadata). Adult specimens were caught with gill nets. Fish were immediately anesthetized with Tricaine mesylate (MS-222) and each fish was identified with an individual code, measured, weighted and photographed in a standardized position for identification. Fish were then euthanized with an overdose of MS-222. The digestive track of each individual was dissected and preserved in separated vials with 100% ethanol and kept at −20°C until processing. Fish were assigned to species in the field according to phenotypic features.

The taxonomic status of the Midas cichlid species in the Nicaraguan lakes is not yet fully resolved. Several Nicaraguan crater lakes hold Midas cichlid populations that are well differentiated from those in the large lakes (Barluenga and Meyer, 2010), but are still taxonomically considered the general type *A. citrinellus*. Likewise, in some crater lakes there are lipped forms somewhat equivalent to *A. labiatus*, but genetically differentiated from the homonymous described species endemic to the large lakes. Waiting for a proper species assignation, *A. citrinellus* and *A. labiatus* from the distinct lakes were here considered as different species. The lipped form from crater L. Apoyeque was considered as a separated morphotype/species and named *A. citrinellus* lipped (Acit_lip) to discriminate it from the common type *A. citrinellus*.

Preserved full digestive tracts were analyzed in the laboratory facilities in Spain. Under sterile conditions, stomachs were separated from intestines according to morphological features described for the convict cichlid (Hopperdietzel et al., 2014). At this stage, potential gut ectoparasites were removed and intestine lengths recorded (Supplementary Table S1). DNA was extracted from individual intestines following the protocol previously described (Baldo et al., 2015) and sent to the Genomics Facility at the Centre for Genomic Regulation (CRG, Barcelona, Spain) for amplicon generation and sequencing. Specifically, the ∼450 bp region encompassing V3–V4 of 16S rRNA gene was amplified in three-replicates with non-barcoded primers S-D-Bact-0341-b-S-17 and S-D-Bact-0785-a-A-21 (Klindworth et al., 2013) with TruSeq adapters. Three mock communities (HM-276D and HM-277D from BEI Resources, and Zymo D6303 from Zymo Research) were included as standard quality control, while nuclease-free water was used as negative control. Successful amplifications from the three replicates were pooled (including water and mock communities) and individually barcoded with a second round of PCR. Amplicons (*N* = 158) were finally pooled in equimolar amounts and cleaned as in Baldo et al. (2015). The same library was sequenced on two separated runs on a MiSeq v3 instrument (600 cycle cartridge, 300 paired-end) to increase sequencing coverage per sample. Raw sequences are available at the Bioproject PRJNA531389.

### Sequence Processing

The same pipeline was used to process raw reads obtained from the two Miseq runs including Nicaraguan cichlids (here named as Midas_run1 and Midas_run2) and from our previous published dataset including African cichlids (Africa_run, available at Bioproject PRJNA341982, comprising 116 samples from two lakes) (Baldo et al., 2017). Both Nicaraguan and African datasets were generated through the same protocol for DNA extractions, amplicon production and sequencing, therefore allowing reliable comparisons. Specifically, paired-end reads from each Miseq run were merged using “make.contigs” and sequences filtered with screen.seqs (maxambig = 0, maxlength = 510, maxhomop = 8) in mothur v.1.25.0 (Schloss et al., 2009). Primers were trimmed with custom R script and chimera removed through vsearch v1.4.4 against the reference gold database (10,362 seqs)^1^. Final number of sequences was 5,234,990 sequences for Midas_run1, 5,655,269 for Midas_run2, and 7,397,114 for Africa_run. Summary of the filtering process is given at Supplementary Table S2.

Sequences from the three runs were then merged into a single FASTA file (18,287,373 total sequences), input into the QIIME pipeline (Caporaso et al., 2010) using Macqiime 1.9.1., and clustered into Operational Taxonomic Units (OTUs) at 97% similarity using pick_open_reference_otus.py with –min_otu_size 10. Taxonomy was assigned with UCLUST against the Greengenes gg_13_8 database with RDP classifier (80% confidence). The matrix obtained was input into R for further filtering according to OTU taxonomy, abundance and frequency across samples. Specifically, (1) only Bacteria were retained, excluding Cyanobacteria, whose classification as purely environmental rather than transiently associated to the host remains to be determined (193 OTUs, corresponding to ∼875,000 sequences largely classified as members of the genus *Synechococcus*), (2) less than three counts per OTU per specimen were considered as zero occurrence, (3) OTUs with less than 50 counts total across cichlids and those occurring in single specimens were discarded, and (4) OTUs detected in the water (*n* = 20, corresponding to 232 reads) were discarded, along those detected in the three mock communities (*n* = 46), except for 11 OTUs, which showed considerable abundance in cichlids (1000 total counts across cichlids and <100 total reads across all mocks).

Representative sequences were aligned with INFERNAL (Nawrocki and Eddy, 2013) in the RDP pipeline^2^. The alignment was trimmed with filter_alignment.py (options g 0.20 and -s to avoid lanemasking) and a phylogenetic tree built with FastTree (Price et al., 2009). Final alignment comprised 402 bp. Two OTU abundance matrices were created: one including all counts (“OTU_ALL”) (available at Supplementary Table S3), and one rarefied to 15,000 counts per sample, therefore discarding nine out of 274 total samples (“OTU_ALL_raref”).

After rarefaction, different abundance matrices were generated for each taxonomic level (phylum to genus, five matrices total) by binning sequence counts by taxonomic rank with the function “aggregate” in the R stats package (R Core Team, 2018). The cichlid core OTUs and taxa were then estimated on all individual matrices using custom R scripts, and calculated as a component shared by at least 90% of the total specimens. We note that because the African and American samples were processed in different laboratories/facilities (except for the sequencing center, which was the same for both projects), potential contamination across the two major datasets can be substantially ruled out.

### Statistical Analyses

Alpha diversity was estimated according to Faith’s phylogenetic diversity (PD whole tree), Shannon and Observed Species on the rarefied dataset (15K), after 10 iterations. Statistical differences in alpha diversity across sample variables were tested with linear mixed effects models, with species nested within lake and lake within continent, with continent as fixed effect. Variance partition was estimated as intraclass correlations calculated from the variances of random terms. To analyze pairwise differences among Midas lakes, a mixed effects model was fit with lake as fixed factor and species within lakes as a random factor. This is a conservative approach, since variance among species was negligible. Pooling observations across species and fitting an ANOVA model only stressed the finding that differences among lakes were driven only by crater L. Apoyo. Linear mixed effects models were also used to test for differences in intestine length and diet. All models were fit with R package nlme (Pinheiro et al., 2017).

Beta diversity was estimated based on Unweighted Unifrac distances and visualized through Principal Coordinates Analysis (PCoA) ordination plots obtained with R function cmdscale in the stats package (R Core Team, 2018). To test for differences in microbiota Unifrac distances across potentially explanatory factors (“continent,” “lake,” and “species”), we used Permutational Multivariate Analysis of Variance (PERMANOVA) (Pinheiro et al., 2017; R Core Team, 2018) for PRIMER v7 (Oksanen et al., 2019). As in the univariate analysis above, species was considered as a factor nested within lake, and lake as nested within continent. We also further considered all factors as fixed. Differences among species within lakes Apoyo and Xiloá were tested by permutational MANOVA with R function adonis in the vegan package (Oksanen et al., 2019).

Microbiota dendrogram was built based on distances between centroids of Unifrac distances per lake [calculated with the usedist R package (Bittinger, 2017)] using the hclust function in the R stats package (R Core Team, 2018). Similarly, a genetic dendrogram was built using average Fst distances by lake based on published microsatellites data (Barluenga and Meyer, 2004). The two dendrograms were compared using the function “tanglegram” in the dendextend R package.

Indicator taxa and OTUs are here defined as those showing significant differences in total sequence counts between lakes and/or continents. For the indicator analyses, the rarefied dataset was additionally filtered to retain OTUs > 500 total counts (retaining 416 OTUs) and >200 counts for the Central American dataset only (retaining 449 OTUs). This further filtering step is crucial to avoid significance for low abundant OTUs. Sequence counts were then aggregated by taxonomic level, creating different abundance matrices, and indicator OTUs and taxa were identified on each matrix with the function indval in the R package labdsv (Roberts, 2016) (cut-off value = 0.70, *p*-value < 0.01).

Venn diagrams were computed with function overLapper in the R package systemPipeR v1.6.2. Heatmaps and most figures were created with ggplot2 (Wickham, 2016) and all subsequent statistical analyses were run in R version 3.4.4 (R Core Team, 2018), except as noted.

## Results

Our final dataset included 274 wild specimens encompassing 42 cichlid species from two African and seven American lakes. After extensive filtering (see section “Materials and Methods” and Supplementary Table S2), we obtained a total of 16,083,249 clean sequences, which were classified to 3639 OTUs (97% identity threshold), 2870 for the Midas cichlid dataset and 2640 for the African cichlid dataset (Supplementary Table S3). These OTUs corresponded to 28 phyla, 73 classes, 132 orders, 177 families, and 200 genera. Overall, 71% of the OTUs could not be classified to the genus level (80% confidence), 27% did not reach the family level, while only 8% remained unclassified to the order level.

### The Cichlid Core Microbiome

**FIGURE 2:**
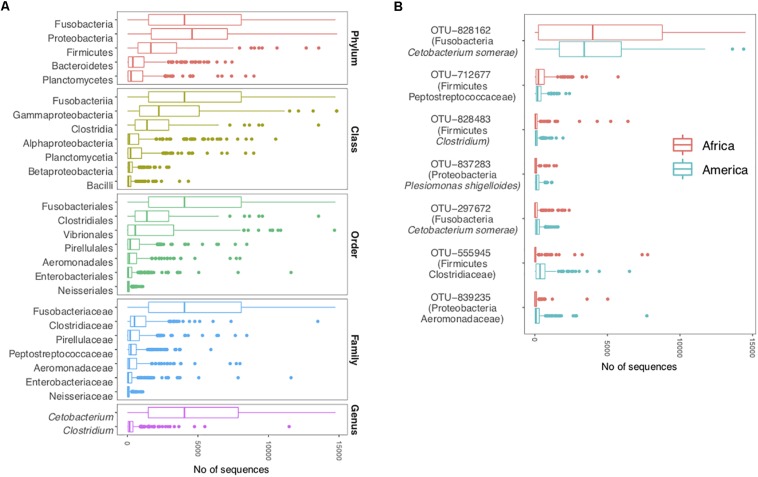
The cichlid core microbiota (each taxon shared by at least 90% of the specimens). **(A)** Core taxa per individual taxonomic levels (from phylum to genus) and overall abundances across all specimens (sequence counts were binned by taxonomic rank). **(B)** Core OTUs and abundances separately for each continent. OTUs are shown along with their taxonomic classification assigned with a confidence ≥80% against the Greengenes database (phylum is followed by the highest taxonomic resolution achieved). Boxplots are centered at the median and whiskers show data dispersion across specimens. The analyses are based on the rarefied dataset (OTU_ALL_raref).

The cichlid core gut microbiota profile, defined as the taxonomic component shared by at least 90% of the specimens and calculated for all individual taxonomic ranks (see “Materials and Methods”), comprised five phyla (Proteobacteria, Fusobacteria, Firmicutes, Bacteroidetes, and Planctomycetes), seven classes, seven orders, seven families, two genera, and seven OTUs (Figure 2). Given the low proportion of OTUs annotated to genus level (29%), additional unknown core genera might exist within the detected core families. Qualitatively, this strict core encompassed a small portion of the total number of taxa detected in the cichlid gut (representing 18% of total phyla, 10% of classes, 5% of orders, 4% of families, 1% of genera, and only 0.2% of total OTUs), while quantitatively, the seven core OTUs alone comprised on average 40% of the total bacterial sequences per specimen (36.5% median). Of the five core phyla, Fusobacteria and Proteobacteria were overall the most abundant and contributed similarly in quantitative terms (median = 4044 and 4579, respectively). Only two genera were almost ubiquitous: *Cetobacterium* and *Clostridium*. The majority of *Cetobacterium* sequences belonged to a single OTU (828162), which was systematically detected in all specimens and classified as *Cetobacterium somerae*. This was also the most abundant OTU in both continents (median across all samples = 3484 sequences), consistently with our previous findings for African cichlids (Baldo et al., 2017), and encompassed almost all sequences associated to the phylum Fusobacteria (Figure 2). According to the best BLASTN hits, OTU-828162 had its best match (100% identity) to the stomach mucus of another cichlid species, *Oreochromis mossambicus*. The other six core OTUs, which occurred at relatively low abundances, typically matched host-associated communities (mostly fish-associated) and/or lake water (100% identity, except OTU-297672 with 99% identity) (Supplementary Table S4).

### Similarities and Differences Between African and American Cichlid Gut Microbiota

**FIGURE 3:**
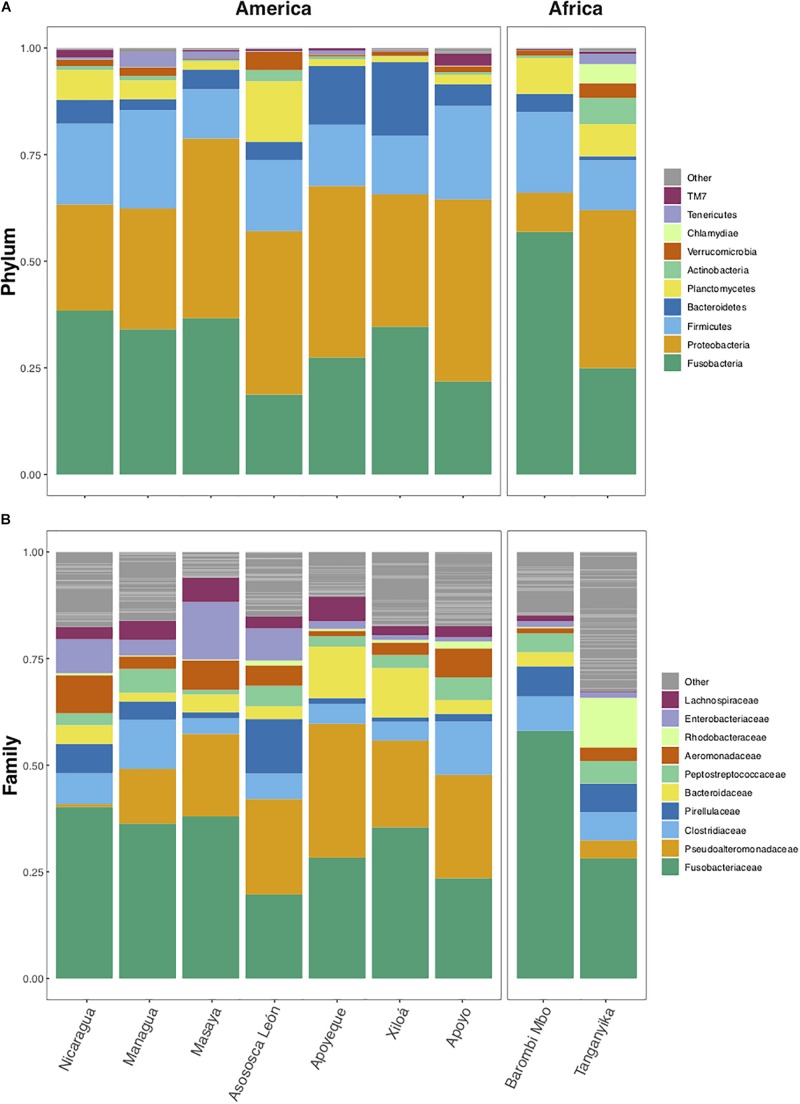
Average relative abundance by lake of bacterial taxa at phylum **(A)** and family **(B)** levels. Dominant taxa are comparable across lakes and continents. Only the 10 most abundant phyla and families are color-coded, while “Others” (gray) include all remaining taxa. The analyses are based on the rarefied dataset (OTU_ALL_raref).

According to the average taxonomic content by lake (Figure 3), the phyla Fusobacteria, Proteobacteria, and Firmicutes were the most abundant in all lakes from both continents (accounting altogether for 80% of total sequences/lake), with Bacteroidetes being predominant over Firmicutes only in crater L. Xiloá (Figure 3A). At the family level, Fusobacteriaceae, Clostridiaceae, and Pseudoalteromonadaceae were highly represented in most lakes, with Pseudoalteromonadaceae being depleted in L. Nicaragua and L. Tanganyika and absent in crater L. Barombi Mbo. Lake Tanganyika was mainly characterized by a higher proportion of Rhodobacteraceae (Figure 3B).

**FIGURE 4:**
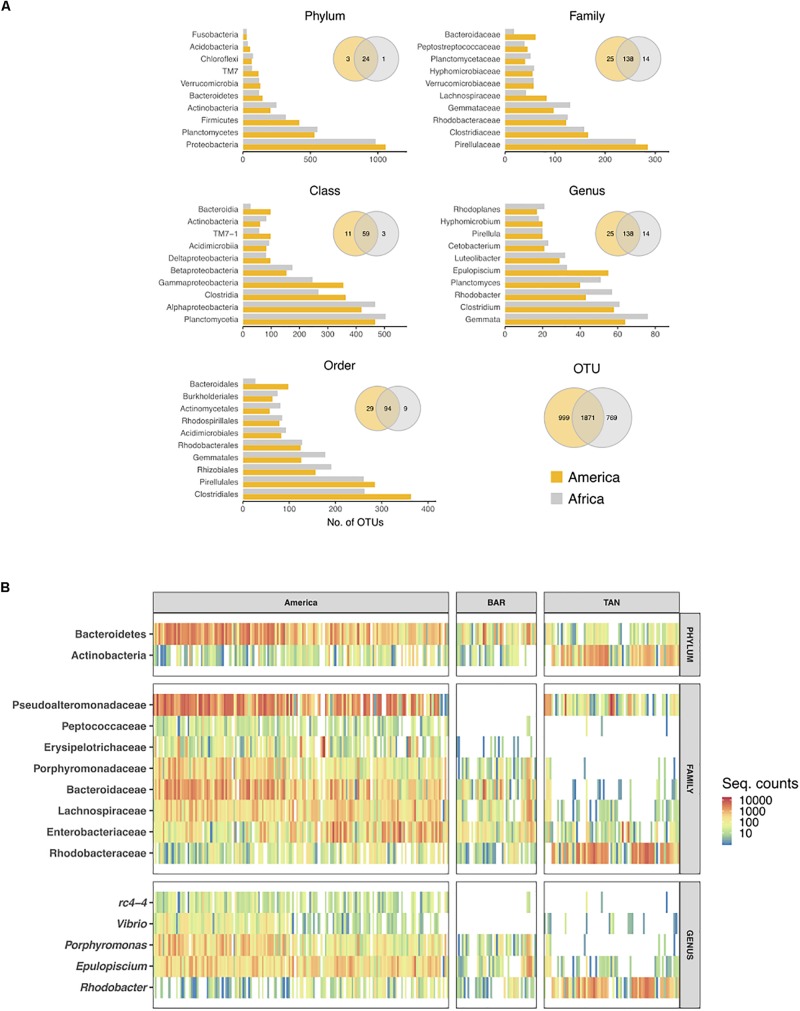
Number of OTUs per bacterial taxon found in each continent **(A)** and heatmap of differentially abundant taxa between continents (ind. value ≥ 0.70, *p* ≤ 0.010) **(B)**. **(A)** Total number of OTUs per taxon were calculated for each taxonomic rank. Only the 10 richest taxa are shown. Venn diagrams show unique and shared taxa/OTUs between continents. **(B)** Colors represent taxon abundance (i.e., sequence counts) per specimen (bar), with white indicating zero counts. For comparative purpose, African specimens were ordered and separated by lake. BAR: Barombi Mbo; TAN: Tanganyika. The indicator analyses are based on the rarefied dataset after additional sequence filtering (see section “Materials and Methods”).

Cichlids from the two continents shared a majority of their microbial taxa, while they were increasingly diverging at higher levels of taxonomic resolution (from phylum to OTUs) (Figure 4A, Venn diagrams). More than half of the total OTUs (51.4% of the 3639 total OTUs) were common to both continental radiations, although the majority of them occurred at low frequency across samples. Likewise, number of OTUs per taxonomic level (from phylum to genus) was highly comparable between continents (Figure 4A, barplots). The phylum Proteobacteria was the richest in both cases, followed by Planctomycetes and Firmicutes, also showing a markedly similar number of OTUs between continents. Proteobacteria diversity corresponded to 1000 total OTUs that encompassed 34 and 38% of total OTUs in the American and African cichlids, respectively. More than half of these OTUs were classified to the class Alpha (Figure 4A), although the class Gamma was quantitatively predominant (Figure 2A). For both continents, most OTUs classified to the phylum Firmicutes belonged to the class Clostridia, order Clostridiales, family Clostridiceae, with the core genus *Clostridium* comprising the largest number of distinct OTUs (∼60). Notably, the phylum Fusobacteria, despite being overrepresented in all lakes in terms of proportion of reads (Figures 2A, 3A), was especially low in number of OTUs (Figure 4A). The most relevant distinction between continents was the number of OTUs associated to the class Bacteroidia, order Bacteroidales and family Bacteroidaceae, up to three times richer in Central American cichlids (Figure 4A).

In terms of indicator values (i.e., those taxa and OTUs showing a significant difference in total sequence counts between continents), Bacteroidetes taxa were significantly enriched in the Central American cichlids (ind. value > 0.70, *p*-value < 0.01 for all taxa/OTUs, Figure 4B). African cichlids were significantly enriched in Actinobacteria, despite this phylum was characterized by a comparable number of OTUs between continents. Quantitative differences in Bacteroidetes and Actinobacteria between continents were clearly driven by L. Tanganyika only (Figures 3, 4B), with crater L. Barombi Mbo resembling more the Central American lakes in quantitative traits. At the family level, Peptococcaceae and Erysipelotrichaceae were virtually absent in the African lakes. The single family Rhodobacteraceae and its associated genus *Rhodobacter* were significantly overrepresented in Africa and particularly in L. Tanganyika. Two genera, *Vibrio* and rc4-4, were a signature of the American samples, being nearly absent in Africa (Figure 4B). A total of 17 OTUs were significantly enriched in the American samples (Supplementary Table S5).

### Alpha Diversity of Gut Microbiota Predicts an Unspecialized Diet in the Midas Cichlids

**FIGURE 5:**
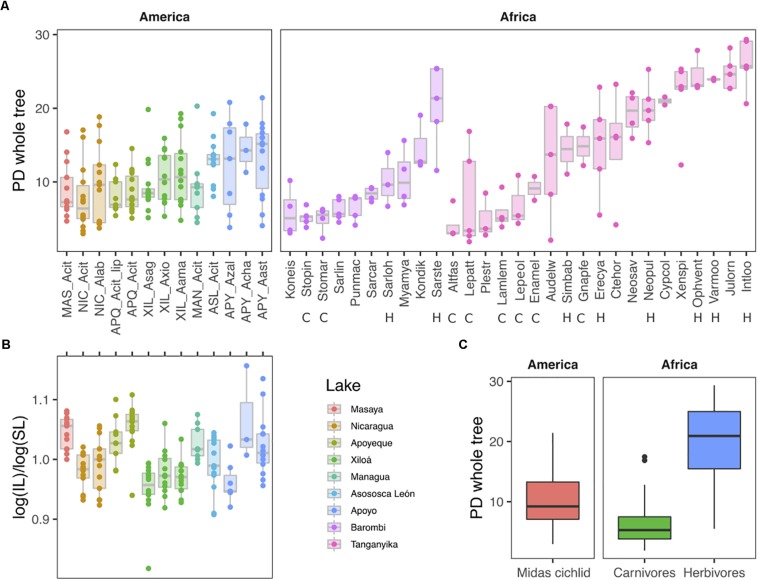
Microbiota Faith’s phylogenetic diversity (PD whole tree) by species **(A)** and continent **(C)** and corresponding intestine morphometrics for Midas cichlids only **(B)**. **(C)** Overall alpha diversity of Midas cichlids (this study) is intermediate between that of African carnivores (C) and herbivores (H) (dataset from Baldo et al., 2017). Dots represent specimens. IL: Intestine lengths; SL: standard body lengths. NIC: Nicaragua; MAN: Managua; MAS: Masaya; ASL: Asososca León; APQ: Apoyeque; XIL: Xiloá; APY: Apoyo. Acit: *A. citrinellus*; Acit_lip: *A. citrinellus* lipped form; Alab: *A. labiatus*; Asag: *A. sagittae*; Axio: *A. xiloaensis*; Aama: *A. Amarillo*; Azal: *A. zaliosus*; Acha: *A. chancho*; Aast: *A. astorquii.* For African species names and diets, see Supplementary Table S1 of this study and Table S1 from Baldo et al. (2017). The analyses are based on the rarefied dataset (OTU_ALL_raref).

Cichlids encompassed a remarkable range of microbial alpha-diversity and richness, with median by species varying up to eight times for the whole tree PD and Shannon indexes, and ∼25 times for number of OTUs (Figure 5A for PD index, and Supplementary Figure S1 for Shannon and Observed Species metrics, all based on rarefied data). According to all three alpha metrics, the least diverse species were the East African scale-eater *Plecodus straeleni* (Plestr) and the carnivore *Altolamprologus fasciatus* (Altfas), while the most diverse species was the East African herbivore *Interochromis loocki* (Intloo) (for African species names and diet see Supplementary Table S1 of this study, for additional information see Table S1 from Baldo et al., 2017). Consistently with their largely omnivorous diet, the Midas cichlids diversity ranged intermediately between the depleted diversity of African carnivores and the extreme one of African herbivores (mixed-effects model, χ^2^(2) = 33.1, *p* < 0.0001) (Figure 5C).

Central American and African cichlids did not differ in their overall measure of PD [χ^2^(1) = 0.28, *p* = 0.596], but variance was partitioned differently in the two continental sample sets. Whereas in African cichlids most of the variation in PD (54%) was attributable to differences between species within lakes, and 25% was attributable to differences between lakes, in Central American cichlids only 15% of the variation was attributable to differences between lakes, and variation among species was negligible. Residual variation (which includes variation between individuals) was only 21% in African cichlids, whereas it accounted for most of the variation in PD (85%) in Central American ones (intraclass correlations from mixed effects models; see also Figure 5A).

Within the Midas species complex, differences among lakes were significant (mixed effects model with lake as fixed factor, χ^2^(6) = 21.625, *p* = 0.0014), but pairwise comparisons revealed that differences were essentially driven by values in crater L. Apoyo, which differed from all other lakes but crater L. Asososca León (Tukey pairwise comparison, *p* < 0.01), while the remaining lakes displayed comparable diversity. Alpha diversity did not differ between benthic and limnetic forms within the two crater lakes where these two forms coexist (L. Apoyo, *F*_1,23_ = 1.53, *p* = 0.106; L. Xiloá, *F*_1,41_ = 1.40, *p* = 0.120).

Microbiota alpha diversity for the Midas cichlid did not correlate with the corresponding normalized intestine lengths for the same set of specimens (*F*_1__,__149_ = 0.04369, *p* = 0.8347; Figure 5B).

### Geographic Factors Have Shaped the Cichlid Gut Microbiota

**FIGURE 6:**
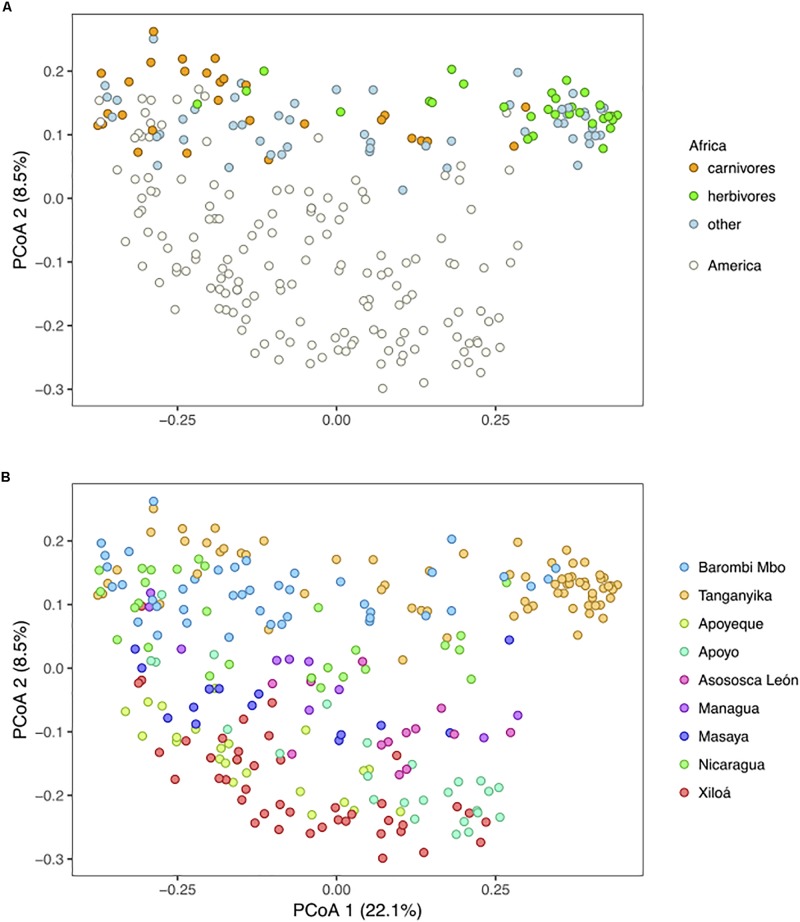
Principal Coordinates Analysis based on Unweighted Unifrac distances depicting clustering by continent and diet **(A)** and by lake **(B)**. **(A)** For the American dataset, major specimen dietary classification is given according to Baldo et al. (2017). PC2 captures the geographic separation between continents and partially among lakes, while PC1 substantially explains variation within lakes, driven by species and/or ecology. Circles represent specimens. The analyses are based on the rarefied dataset (OTU_ALL_raref).

All three major factors considered, “continent,” “lake,” and “species,” significantly explained part of the cichlid microbiota variation (PERMANOVA *p* < 0.001, Supplementary Table S6). According to PCoA based on Unweighted Unifrac distances, PC1, which captured most of the variance (22.1%), indicated important within-lake differences, while the geographic signal was substantially recovered by PC2, which ordered the samples by continent and partly by lake (Figure 6A). For the two African lakes, which are relatively species-rich and show strong dietary specialization, the within-lake differences were mostly associated to variation along the range of dietary variation (with herbivores occupying the far right of PC1) as previously shown (Baldo et al., 2017). Interestingly, the range of Midas cichlids variance along PC1 was contained within that of both African carnivores and omnivores, while African herbivores clustered apart (Figure 6A). For the Midas cichlids, which are relatively species-poor and belong to a unique genus (i.e., *Amphilopus*), the microbiota did not significantly differ across species within lakes, not even in the two crater lakes with most ecologically diverse species, lakes Apoyo and Xiloá (adonis, *p* > 0.05 for both lakes in comparisons across benthic and limnetic forms; Supplementary Figure S2). This indicates that the large Midas cichlid microbiota spectrum along PC1 (Figure 6A) was mostly driven by intraspecific variance, with an important overlap among species and ecological niches.

### Microbial and Genetic Distances Infer Comparable Relationships Among Midas Cichlids

**FIGURE 7:**
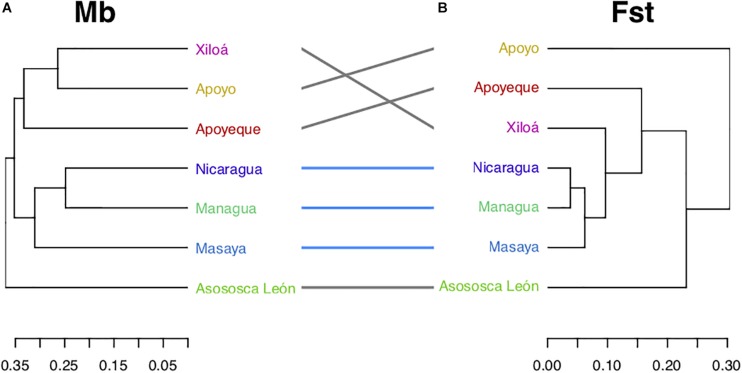
Midas lake cichlid relationships as inferred by their microbiota (Mb), and Fst genetic distances. **(A)** Microbiota dendrogram according to the lake centroid by mean of Unifrac distances among specimens, **(B)** dendrogram based on average microsatellites-based Fst distances among lakes from previously published data (Barluenga and Meyer, 2010), largely in agreement with SNPs-based data from Kautt et al. (2018). Common labels (lakes) are connected by lines, whereas lakes in common subtrees are connected by blue lines. The cluster of large lakes (Nicaragua and Managua) with the crater L. Masaya was recovered by both distances.

To explore the cichlid microbiota community pattern among the Nicaraguan lakes, microbiota Unifrac distances were collapsed using the centroids across all individuals within each lake (Figure 7A) and compared to the cichlid genetic distances among lake assemblages as inferred by published microsatellites data (Figure 7B) (Barluenga and Meyer, 2010), overall supported by recent SNPs data (Kautt et al., 2018). Major microbiota-based clustering was largely congruent with genetic-based inferences, both supporting (i) the grouping of the two large lakes (L. Nicaragua and L. Managua) and crater L. Masaya, (ii) the close resemblance between crater L. Xiloá and crater L. Apoyeque, and (iii) the distinctness of crater L. Asososca León from all other lakes. The major discordance occurred with crater L. Apoyo, similar in microbiota to crater L. Xiloá, while their fish are genetically divergent (Figure 7).

We next explored the microbial components supporting the above-observed microbiota clustering among lakes, focusing on patterns of dissimilarities (driven by discriminatory OTUs/taxa) and similarities (driven by shared OTUs/taxa) as seen at different scales (lake, species and trophic niche).

### Midas Cichlid Core Microbiota and Indicator Values

**FIGURE 8:**
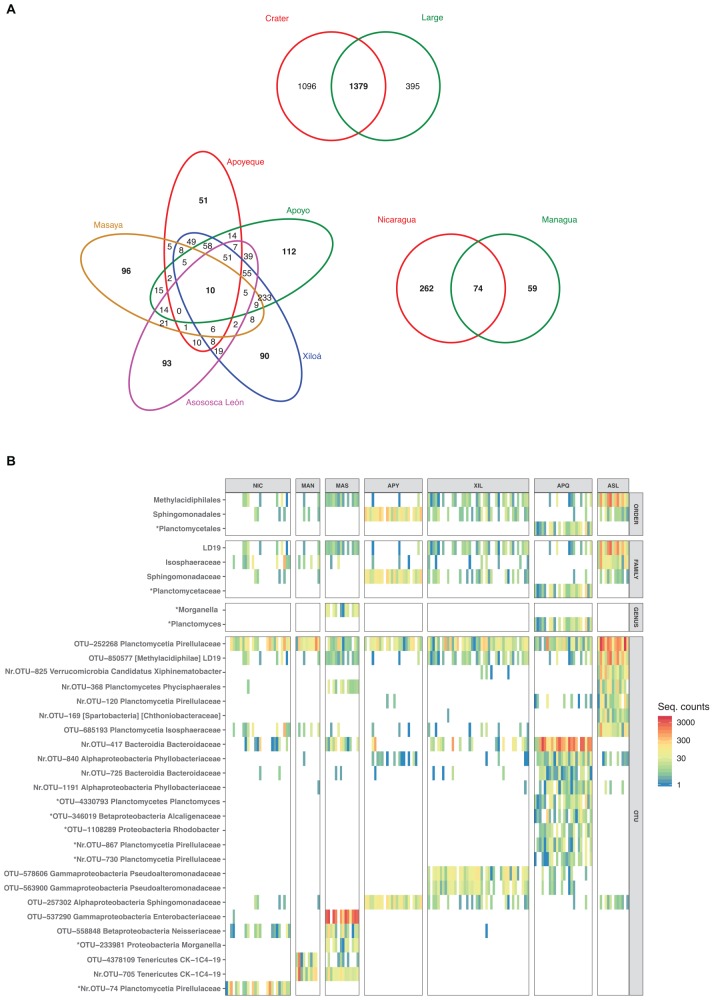
Midas cichlid shared and unique OTUs **(A)** and heatmap of differentially abundant taxa and OTUs **(B)** (ind. value ≥ 0.70, *p* ≤ 0.010). **(A)** Venn diagrams display shared and unique OTUs between large and crater lakes (top), among the five crater lakes (left, only crater-specific OTUs) and between the two large lakes (right, only large-specific OTUs). Total number of OTUs per lake was: Nicaragua = 433; Managua = 998; Masaya = 1018; Asososca León = 1102; Apoyeque = 812; Xiloá = 1423; Apoyo = 1456. **(B)** Only taxonomic levels with significant values (order, family, genus and OTUs) are shown. Colors represent taxon abundance (i.e., sequence counts) per specimen (bar), with white indicating zero counts. OTUs are shown along with their taxonomic classification assigned with a confidence ≥80% against the Greengenes database (phylum is followed by the highest taxonomic resolution achieved). Squared brackets indicate uncertain classification. Nr: New Reference OTU. The indicator analyses are based on the rarefied dataset after additional sequence filtering (see section “Materials and Methods”).

At the qualitative level, 7% (198 OTUs) of the total OTUs from the Midas cichlids (2870) were found in all lakes. The large lakes and the crater lakes shared almost half of their total OTUs (48%, corresponding to 1379 OTUS, Figure 8A); 74 OTUs were exclusive of the two large lakes, while 10 OTUs were exclusive of all the crater lakes. After including crater L. Masaya with the large lakes, according to their closer resemblance (Figure 7), the number of crater-lake core OTUs increased from 10 to 51 (Supplementary Figure S3).

Lake-specific OTUs represented only a small fraction of the total microbiota, varying between 6% (crater L. Apoyeque) and 18% (L. Nicaragua) (Figure 8A). Crater L. Apoyeque displayed the lowest number of unique OTUs (51, corresponding to 6% of its total OTUs, Figure 8A), sharing the majority of them with the geographically nearby crater L. Xiloá. Crater lakes Apoyo and Xiloá shared the largest fraction of OTUs overall (Jaccard distance = 0.41), with 16% of their total OTUs being uniquely found in these two lakes (233 OTUs). This pattern alone largely explains the close microbiota resemblance among these three lakes (Apoyeque, Apoyo and Xiloá) as inferred by average Unifrac distances (Figure 7A). Crater L. Asososca León displayed the highest percentage of unique OTUs among crater lakes (8.4%, corresponding to 93 OTUs), excluding crater L. Masaya (9.4%).

Likewise, the pattern of relative abundance of taxa and OTUs by lake among Midas cichlids clearly supported some of the major Unifrac groupings. Specifically, only a few significant indicator values (ind. value > 0.70, *p*-value < 0.01 in all cases) drove the separation between the large lakes and the crater lakes, including an enrichment in *Plesiomonas shigelloides* in the large lakes, and in the order Vibrionales, genus *Vibrio* in the crater lakes (five OTUs, all classified as *Vibrio cholerae* with 100% identity according to best BLASTN hits), along with several OTUs of the family Pseudoalteromonadaceae (Supplementary Table S5).

Only few taxa showed significant quantitative differences among lakes, and these differences were typically driven by single OTUs (Figure 8B, asterisks indicating lake-specific OTUs/taxa). Cichlids from crater lakes Apoyeque and Asososca León were the most differentiated, showing the highest number of significant indicator taxa and OTUs, while L. Nicaragua was the least distinguished, with a single enriched OTU (see also Supplementary Table S5) (ind. value > 0.70, *p*-value < 0.01 in all cases). More specifically, crater L. Asososca León showed a higher abundance of the order Methylacidiphilales and its representative family LD19 (OTU-850577). Crater L. Apoyo was significantly enriched in the order Sphingomonadales and its representative family Sphingomonadaceae (OTU-257302). L. Apoyeque was characterized by a unique enrichment in the order Planctomycetales, genus *Planctomyces* (OTU-4330793), whereas L. Masaya fishes showed the unique enrichment in the human pathogenic bacterium *Morganella morganii* (OTU-233981) and in the Enterobacteriaceae OTU-537290 (Figure 8B). Within the 25 significant indicator OTUs detected, nine were lake-specific (with asterisks in Figure 8B); of these, four were found exclusive of L. Apoyeque and included members of the Pirellulaceae and Alcaligenaceae families, as well as two OTUs classified to the genera *Rhodobacter* and *Planctomyces*. Within the 25 significant indicator OTUs detected, nine were lake-specific; of these, four were found exclusive of L. Apoyeque (Figure 8B).

We finally compared patterns of microbial abundance as a function of the Midas cichlid ecological niches, focusing on the well-studied comparison between benthic and limnetic forms within crater lakes Xiloá and Apoyo. In both lakes, the two benthic species (shallow and deep) showed a higher number of indicator taxa compared to the limnetic species (Supplementary Table S7). However, no niche-specific OTUs/taxa were common to the equivalent ecotypes from the two lakes. In crater L. Xiloá, the benthic deeper species (*A. xiloaensis*) was enriched in Verrucomicrobia and particularly in the species *Akkermansia muciniphila*; the benthic shallow species (*A. amarillo*) was enriched in the orders Chromatiales and Rhizobiales and family Peptostreptococcaceae; the limnetic species (*A. sagittae*) did not show any significant enrichment. In crater L. Apoyo, the benthic deeper species (*A. chancho*) was enriched in Actinomycetales and an OTU classified to the family Lachnospiraceae; the benthic shallow species (*A. astorquii*) was enriched in Ruminococcaceae; the limnetic species (*A. zaliosus*) was enriched in two OTUs classified to the family Ruminococcaceae (Supplementary Table S7).

## Discussion

### A Unifying Cichlid Gut Microbial Signature

Cichlids harbor a largely similar gut microbiota, in terms of major compositional traits, over the large range of geographic and ecological diversity considered (Figures 1–3). The detection of a core set of bacterial taxa found in >90% of the specimens analyzed across continents, lakes, species and very contrasting dietary niches (Figure 2), supports a fundamental role of these taxa in structuring the cichlid gut microbiota. At phylum level, this “minimum” core appears as rather common among fishes, with Proteobacteria, Firmicutes, and Bacteroidetes typically representing the major microbial component of both freshwater and marine fish guts (Sullam et al., 2012; Egerton et al., 2018). Yet, the prevalence of Fusobacteria in the cichlid gut seems to be rather specific, as this phylum is usually found as a minor constituent in other fishes (Sullam et al., 2012; Sevellec et al., 2018).

While the core microbial taxa encompass only a subset of the maximum diversity observed in a cichlid gut, its cumulative abundance contributes to a much greater extent (Figure 2, median of 36.5% of sequences per sample), a pattern that was also recently found for lake whitefish (Sevellec et al., 2018). Notably, these core OTUs also displayed quite comparable abundances between continents, supporting the idea of a conserved host-microbes and microbes-microbes physiological regulation for this putatively host-adapted core (Goodrich et al., 2014; Shapira, 2016). A single OTU, classified as *C. somerae*, stood out as the predominant component of the cichlid gut microbiota (Figure 2), as also consistently reported in our previous studies (Baldo et al., 2015, 2017). This species is putatively capable of producing vitamin B12 (Tsuchiya et al., 2008), an essential key nutrient for fish, and was formerly detected as a core species in other freshwater fish species (Roeselers et al., 2011; Sullam et al., 2012, 2015). Members of the genus *Clostridium* (here represented by one or possibly two core OTUs, Figure 2) were also repeatedly found as core taxa in other fishes (Egerton et al., 2018; Sevellec et al., 2018). While some bacterial genera might be simply very widespread, the systematic presence of specific OTUs in the gut of geographically distant and isolated lineages (as those found in distinct lakes) are most likely associated to a certain level of host-specificity and putative restricted transmission (Wang et al., 2018). However, due to the lack of data from taxa other than cichlids within the studied lakes that could act as “control” (sampling is currently ongoing), it remains unclear at a moment whether these core OTUs are cichlid-specific, fish-specific, or simply globally widespread opportunist bacteria able to colonize the fish gut (Egerton et al., 2018). The introduction of the African Tilapia from aquaculture into several Nicaraguan lakes (Mccrary et al., 2007), along with their “captive” gut microbial communities, is unlikely to explain the microbial similarities observed across Central American and African cichlid radiations, given that Tilapia has not reached all lakes, and that captivity has been shown to have a dramatic effect on the cichlid gut microbiota composition (Franchini et al., 2014; Baldo et al., 2015).

Overall, the existence of stable microbial features over a broad spectrum of cichlid diversity supports a major role of the host selective constraints, here underlined by the cichlid common genomic background (Brawand et al., 2014), in the recruital, assembly and maintenance of a “cichlid” gut microbiota, as increasingly shown in other vertebrate species (Roeselers et al., 2011; Seedorf et al., 2014; Sevellec et al., 2018). Contribution and mechanisms of transmission modes (vertical or cycling) to the propagation and maintenance of gut bacteria in cichlids remain to be quantified, along with their functionality.

### Geographic Isolation Represents the First Level of Cichlid Gut Microbiota Structuring

Within a restricted number of bacterial phyla, cichlid fish intestines can potentially accommodate a remarkably diverse ensemble of microbial taxa, as shown by their great range of alpha and beta diversities (Figures 5, 6). Their lake and continental separation clearly accounts for part of this variance, providing the first level of discrimination across microbial communities. Concurrent host-specific and environmental factors can both be responsible for this spatial/temporal variance, including timing of cichlid isolation events into distinct water bodies, as well as exposure to lake-specific biotic and abiotic parameters.

African and Central American cichlid radiations diverged most likely after the continental drift, with a trans-Atlantic dispersal of cichlids estimated between 89.4 and 74.0 Mya, or earlier (Matschiner et al., 2016). Despite this large spatial and temporal segregation, fishes from the two continents shared, on average, most microbial taxa (up to genus level, with only 10 and 19% of genera being continent-specific) and a comparable OTU diversity per taxon (Figure 4A), with only few differentially abundant taxa (Figure 4B). Dissimilarity was mainly driven by the microbial composition of cichlids from L. Tanganyika, reservoir of some of the oldest cichlid lineages, and currently hosting the greatest diversity of species in terms of genetics, morphology and ecology (Salzburger et al., 2002, 2005). Cichlids from L. Tanganyika harbored a significantly higher abundance of Actinobacteria, along with a conspicuous enrichment in the family Rhodobacteraceae, while being depleted in Bacteroidetes (Figures 3, 4B). Such uniqueness in microbiota structure clearly stems from the highest levels of trophic specialization found in this lake; enrichment in Actinobacteria and Rhodobacteraceae was previously detected as a signature of true herbivory, a trophic niche exclusive of few Tanganyikan cichlid species (Baldo et al., 2017).

Biochemical aspects of the lake water, including variation in water salinity and presence of other chemical compounds, can also represent major selective parameters driving diversity in the fish gastrointestinal physiology (Wong et al., 2014) and associated microbial communities (Schmidt et al., 2015; Wang et al., 2018). Indeed, levels of salinity in the Nicaraguan lakes are known to vary to a great extent, particularly between the large lakes and the crater lakes (Barloy et al., 1976), potentially explaining part of the microbial diversity seen among their cichlid assemblages. Moreover, the presence of a distinct community of fishes (besides the ones under study) and invertebrate species among lakes can also drive variation in the surrounding microbial ecosystem, exposing fishes to variable sources of bacteria through diet and water ingestion, two important factors in shaping the fish gut microbiota (Bolnick et al., 2014; Giatsis et al., 2015; Miyake et al., 2015; Egerton et al., 2018). The lack of characterization of the environmental microbiota for the lakes under study (which is currently ongoing) precludes further speculations in this respect, although water has been typically found to have a minor contribution to the fish gut microbiota (Giatsis et al., 2015; Sevellec et al., 2018).

Interestingly, a few putative pathogens were found to account for some of the differences among Nicaraguan lakes, including members of the genus *Vibrio*, mostly identified as *V. cholerae* and enriched in crater lakes, and *M. morganii*, recovered in most specimens from the highly polluted crater L. Masaya. Both freshwater and marine fishes have been found to host *Vibrio* species (Egerton et al., 2018) and *V. cholerae* (Halpern and Izhaki, 2017), while incidence of *M. morganii* has been previously reported in various fishes (Kim et al., 2000; Reshma et al., 2018), suggesting in both cases that the fish gut might represent an important reservoir for the global dissemination of these putatively opportunistic human pathogens.

### The Gut Microbiota of the Midas Cichlid Harbors a Phylogeographic Signal

For the young radiations of the Midas cichlid species complex, where lineage differentiation is not extensive (encompassing a single genus, *Amphilophus*), and ecological variation is limited (all fish are mostly omnivorous), average microbiota distances among lakes can be explored as a function of lake connectivity (considering historical and present water connection among lakes, and potential translocations of cichlids or other animals across lakes), and cichlid phylogeographic history (including timing of colonization of the crater lakes, strength of founder effect and other demographic aspects) (Barluenga and Meyer, 2010; Elmer et al., 2010; Kautt et al., 2018).

Timing and trajectories of the colonization events of Nicaraguan lakes by the Midas cichlid, as well as processes involved in the subsequent sympatric diversification within lakes have been largely resolved with genomics (Barluenga and Meyer, 2010; Kautt et al., 2018) and morphological data (Elmer et al., 2010, 2014). These data provide the historical scaffold to explore how the gut microbial communities have changed along this young cichlid radiation. We found that the average microbiota distances among lakes (according to Unweighted Unifrac) partly matched the genetic distances of their corresponding cichlid hosts (Figure 7). The major congruency observed was the grouping of the two large lakes, once physically connected, and still today intermittently connected through the R. Tipitapa, potentially exchanging fish and water along with their microbial communities. Interestingly, and in accordance to fish genetics, the large lakes also resemble in microbial signature to crater L. Masaya. This crater lake offers a very distinct environment with respect to the two large lakes (small size with deep layered waters), suggesting that the observed resemblance of their cichlid gut communities is due to a putative faunal exchange rather than being driven by common environmental/ecological parameters.

Among the other crater lakes, representing isolated water bodies with overall comparable ecological and biogeochemical conditions, cichlids from the smallest and most isolated crater L. Asososca León (located on the north-west of L. Managua, Figure 1) hosted the most discriminated microbiota (Figure 7A), as supported by the presence of several indicator values (Figure 8B). This finding is in agreement with genetic and historical data indicating that L. Asososca León might be the oldest crater lake, colonized from the source L. Managua around 1550 generations ago (Kautt et al., 2018). This lake hosts a genetically well-distinguished cichlid assemblage (with 100% of mtDNA private alleles) (Barluenga and Meyer, 2010), and might still retain part of the ancestral genetic pool from the original source population. Such genetic distinctness is not accompanied by pronounced morphological differences (Barluenga and Meyer, 2010) and intestine lengths are in the range of those found in the large lakes (Figure 5B). Some environmental features of this lake appear to be unique, such as parasite communities and water chemistry (MB unpublished data), suggesting that the unique features of the cichlid gut microbial communities could be due to lake-specific features.

Consistently with their close genetic distances, the cichlid assemblages in L. Apoyeque and L. Xiloá also shared resembling gut microbiotas (Figure 7), suggesting fish introgression events between these two lakes. Although these lakes are in close proximity [their rims being only 700 m apart (Kautt et al., 2018)], the colonization by fish of crater L. Apoyeque remains unexplained due to its isolation and challenging accessibility.

The major incongruence we detected between microbiota and genetic data was found in the position of cichlids from crater L. Apoyo, which are genetically well differentiated from all other lakes cichlids, but showed microbial communities with overall resemblance to cichlids from crater L. Xiloá (with 233 OTUs being uniquely found in these two lakes). The Midas cichlids of the two lakes, which are geographically distant (Figure 1), originate from different source populations (the northern crater L. Xiloá was seeded by L. Managua, while the southern crater L. Apoyo was seeded by L. Nicaragua), and historical records indicated no events of lake water connection or fish population admixture (Barluenga and Meyer, 2010; Kautt et al., 2018), leaving these microbial similarities (up to OTU level) currently unexplained.

A recent work by Kautt et al. (2018) suggested that all Nicaraguan crater lakes were initially seeded by a small number of individuals, all stemming from populations in the large lakes, which underwent a rapid demographic expansion. Despite this strong founder effect and expected stochastic loss of part of the original microbial pool following host bottlenecks and genetic drift (Baldo et al., 2018), gut microbiota diversity and composition among Nicaraguan cichlids are largely comparable at both qualitative and quantitative levels, with a small number of OTUs being universally found in all crater lakes (51, when excluding L. Masaya, see Supplementary Figure S3). While this microbial trait conservation might be driven by the fish close genetic relatedness, the recent suggestion of a secondary wave of colonization from the source population into all crater lakes (Kautt et al., 2018) could also be playing a relevant homogenizing role. Nonetheless, at a more global scale (thus including African cichlids), it is apparent that less stochastic processes are at play in maintaining a cichlid common microbial signature in the gut.

### Within-Lake Diversification of the Gut Microbiota Is Mostly Associated to the Level of Cichlid Niche Overlap

Whereas the cichlid physical separation in distinct water bodies has undoubtedly driven variation in their gut microbiota, with levels of lake connectivity and isolation timing (as inferred by their genetic distances) explaining relationships across lakes, variance within lakes can be higher than among lakes for both alpha and beta diversities, pointing to important within-lake trends of microbiota diversity (Figures 5, 6). This is especially relevant for sympatric species that are confined to a restricted environment, such as crater lakes. In this respect, particularly interesting is the comparison between the Nicaraguan crater lakes and the African crater L. Barombi Mbo: unlike Nicaraguan large lakes and the Great L. Tanganyika, African and American crater lakes are comparable in size and overall environment (Figure 1), with species having segregated along a depth axis, although the time of the radiations is different. The sympatric radiation of crater L. Barombi Mbo is 0.5–1 My old and currently hosts a relatively larger number of cichlid species (*n* = 11) that have differentiated into multiple genera characterized by strong dietary specializations (Trewavas et al., 1972), whereas the oldest cichlid assemblage from the Midas cichlid in the crater lakes (excluding L. Masaya) dates back to 0.02 My (therefore being at least 25 times younger), and speciation remains incipient, with an ecological and phylogenetic diversity comparably lower (Barluenga and Meyer, 2010; Kautt et al., 2016a, b, 2018). At the microbiota level, these differences are reflected on a stronger discrimination among species in crater L. Barombi Mbo than in any of the Midas cichlid crater lake populations, both in terms of alpha (Figure 5A) and beta diversities (Figure 6) (Baldo et al., 2017).

At the alpha-diversity level, the Midas cichlid showed a comparable pattern among species within lakes, and typically between lakes (except for crater L. Apoyo being significantly more diverse) (Figure 5A), a finding that is compatible with their substantially equivalent dietary habits, being all largely omnivorous. Conforming to our expectations, the Midas cichlid alpha diversity did not show either the extreme diversity of African herbivores or the depauperated diversity of African carnivores (Figure 5C), reflecting their lack of dietary specialization. As previously proposed (Baldo et al., 2017), these findings support the comparative use of microbiota alpha diversity measures in approximating the cichlid dietary niche, particularly for discriminating between generalist and specialized feeders and, within the latter, between carnivores and herbivores.

At the beta-diversity level, no significant differences were found among species/trophic niches within Nicaraguan lakes. This general lack of microbiota delineation among sympatric species within the Nicaraguan lakes contrasts with the significant microbiota structuring in the more derived species from the African lakes, particularly from crater L. Barombi Mbo (although number of sampled specimens per species was more limited in this case), and appears associated to the distinct age of the radiations. The Midas cichlid is characterized by a “speciation continuum,” with new forms partially overlapping in trophic niches and still incomplete reproductive barriers, as supported by ongoing hybridization (e.g., in crater L. Xiloá) (Kautt et al., 2016a). These processes can help maintaining the gut microbiota connectivity among sympatric species/morphotypes. Nonetheless, we did find few taxa/OTUs among species within crater lakes Apoyo and Xiloá, both of which conform to the classical model lakes for sympatric speciation, suggesting that their gut microbial communities are diverging, along with their host niche segregation. A recent study by Sevellec et al. (2018) on dwarf and normal whitefish pairs (*Coregonus clupeaformis*) from different lakes, representing limnetic and benthic species respectively, provide an interesting comparative study system for gut microbiota diversification in closely related sympatric species/morphotypes. Concordant with our findings in cichlids, also in this case the lake effect was a major factor in shaping the gut microbiota, while the two sympatric morphotypes did not significantly discriminate in either alpha or beta diversities. Potential host genetics effects and partial niche overlap could also play a relevant homogenizing role in this system by increasing microbial connectivity. Further quantitative studies, focusing on a in-depth characterization of the trophic niche overlap and genetic flux among cichlid species, accompanied by a characterization of the environmental and non-cichlid microbiota, are necessary to understand the processes underlying the host intra and inter-specific variance in these sympatric systems.

## Conclusion

The comprehensive dataset analyzed allows extrapolating some fundamental patterns in the cichlid-gut microbiota association in nature, which can inform on general trends in other fish systems. Our findings indicate that multiple concomitant factors affect the cichlid/gut bacteria symbiosis. On the one side, the cichlid genetics likely shapes the basic pattern of microbiota composition, largely unifying the gut community structure profile (both qualitatively and quantitatively) across a broad range of cichlid variation: the most abundant taxa (mainly phylum to family) are consistently the same across all cichlids, together with fewer core taxa/OTUs that occur systematically. Future experimental manipulation of the cichlid gut microbiota by means of transplants could help corroborating this indirect observation and quantify the impact of cichlid genetics. Within this partly constrained “cichlid” microbiota profile, the microbial variation observed across specimens/species appears to be mainly a function of the level of connectivity of their gut communities (chances of contact) and the strength of host-specific selection, mostly driven by niche-associated and environmental parameters. In allopatry, geographic isolation and thus lack of microbial connectivity is a major determinant of the microbiota structure. At the same time, ecological similarity can drive microbiota similarity in allopatric species (as in the case of crater L. Barombi Mbo and L. Tanganyika) in virtue of comparable host selective pressures on bacteria retention from a heterogeneous environmental microbial pool (driven, for instance, by similar dietary requirements, such as in the case of strict herbivory). In sympatry, where microbial connectivity among species is favored by the sharing of the same water pool (fish feces are constantly washed out along with their microbial content), patterns of microbiota similarity/differences among cichlid species are likely a function of their level of trophic niche overlap, habitat segregation and reproductive barriers (the latter being correlated with their phylogenetic relatedness). Finally, the contribution of inheritance of gut bacteria to the cichlid microbiota structure by means of direct or indirect transmission remains to be addressed. Overall, for a true understanding of the eco-evolutionary dynamics of the symbiosis, we highlight the need for an integrative ecosystem network study that includes host-microbes-environment interactions.

## Data Availability Statement

The datasets generated and/or analyzed during the current study are available in at Bioproject PRJNA531389 (America dataset, this study) and PRJNA341982 [Africa dataset (Baldo et al., 2017)].

## Ethics Statement

Specimen sampling and manipulation were approved by the Ministry of the Environment and Natural Resources of Nicaragua (MARENA, permit No. 001-012015 to MB). This study was carried out in accordance with the principles of the Basel Declaration and recommendations of ARRIVE guidelines issued by the NC3Rs, with approval from the Comissió de Bioètica (CBUB) of the University of Barcelona (Spain).

## Author Contributions

LB and MB conceived the study. LB performed the laboratory assays, analyzed the data, and wrote the manuscript. JR performed most of the statistical analyses, wrote corresponding methods, and helped in the data interpretation. MB collected the fish in Nicaraguan lakes and helped with the data interpretation. WS collected the fish in L. Tanganyika. All authors edited and approved the final version of the manuscript.

## Conflict of Interest

The authors declare that the research was conducted in the absence of any commercial or financial relationships that could be construed as a potential conflict of interest.

## Footnotes

1 https://drive5.com/uchime/uchime_download.html

2 https://pyro.cme.msu.edu/login.spr

## Supplementary Material

FIGURE S1 Microbiota alpha diversity by species based on Observed Species (Number of OTUs) and Shannon.

FIGURE S2 Principal Coordinates Analysis based on Unweighted Unifrac distances depicting comparisons across the three species/ecological niches in L. Xiloá and L. Apoyo. The centroids by species are shown. No significant differences among species were found (Adonis, *p* > 0.05).

FIGURE S3 Venn diagrams showing shared and unique OTUs **(A)** between large lakes and L. Masaya versus the other crater lakes, **(B)** among crater lakes excluding L. Masaya (889 total OTUs), and **(C)** among L. Managua, L. Nicaragua and L. Masaya (674 total OTUs).

TABLE S1 Sample metadata.

TABLE S2 Filtering process in mothur.

TABLE S3 OTU_ALL abundance matrix, including the 3639 OTUs and sequence counts by specimen.

TABLE S4 Best BLASTN hits of the seven core OTUs (shared by >90% of the specimens).

TABLE S5 Discriminatory taxa and OTUs between continents and across lakes.

TABLE S6 PERMANOVA results.

TABLE S7 Discriminatory taxa and OTUs among species and trophic niches within crater lakes Apoyo and Xiloá.
