# Erratum: Phylogeography and Ecological Niche Shape the Cichlid Fish Gut Microbiota in Central American and African Lakes

**DOI:** 10.3389/fmicb.2020.00567

**Published:** 2020-03-24

**Authors:** Frontiers Production Office

**Affiliations:** Frontiers Media SA, Lausanne, Switzerland

**Keywords:** allopatry, sympatry, Mida cichlid, continent, Illumina 16S rRNA, *Amphilophus spp*

Due to a production error, there was a mistake in Supplementary Figure 1. The wrong figure was published. The corrected figure has been updated in the Supplementary Material.

The original article has been updated.

