# Supplementary material for: Phylogeography and Ecological Niche Shape the Cichlid Fish Gut Microbiota in Central American and African Lakes
Source: Front Microbiol. 2019 Oct 15;10:2372. doi: 10.3389/fmicb.2019.02372 (PMC6803461; doi:10.3389/fmicb.2019.02372)
Supplement: FIGURE S1 — Microbiota alpha diversity by species based on Observed Species (Number of OTUs) and Shannon. [file Image_1.pdf]

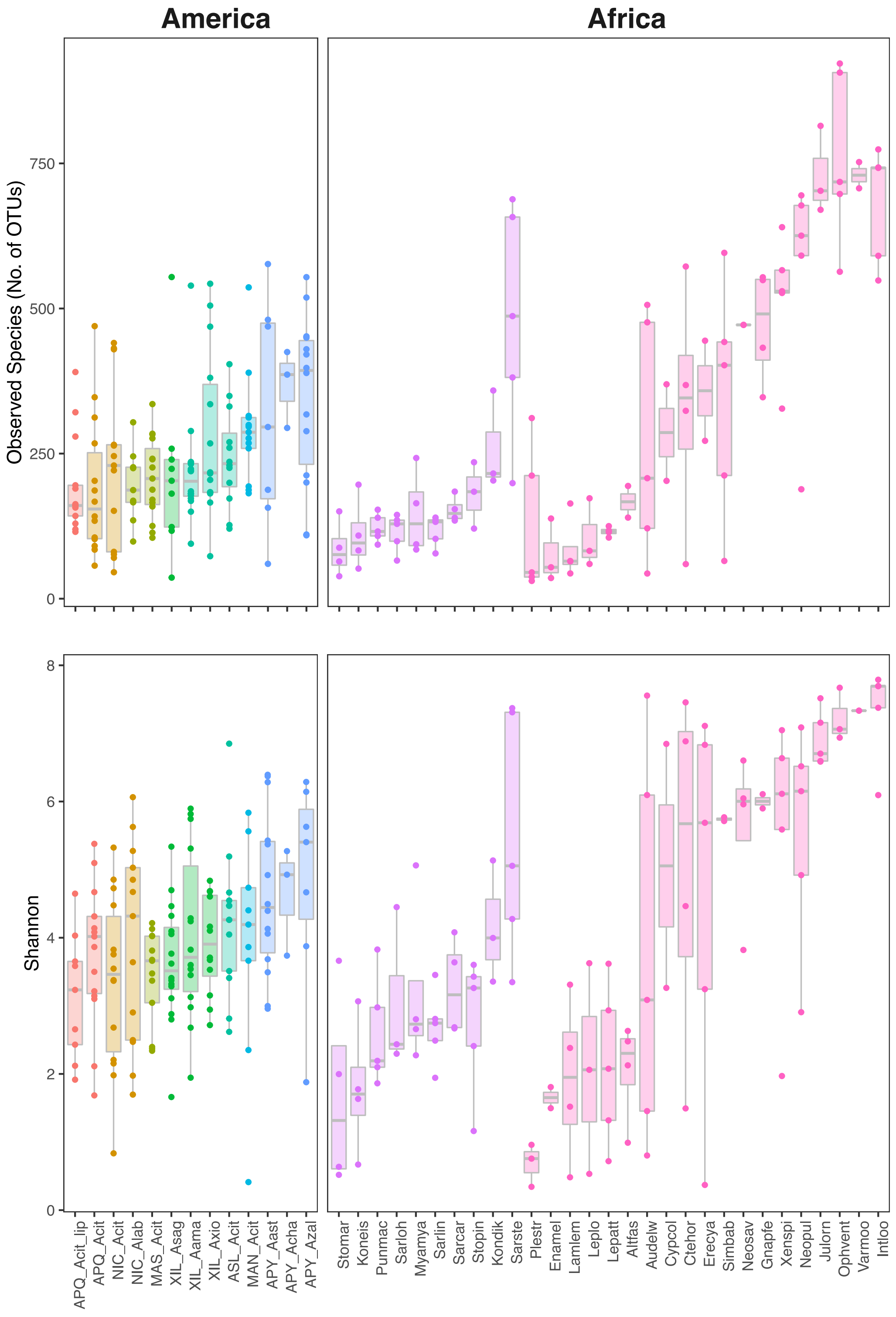

Figure S1: Microbiota alpha diversity by species based on Observed Species (Number of OTUs) and Shannon diversity.
