# Supplementary figures and images for: Phylogeography and Ecological Niche Shape the Cichlid Fish Gut Microbiota in Central American and African Lakes
Source: Front Microbiol. 2019 Oct 15;10:2372. doi: 10.3389/fmicb.2019.02372 (PMC6803461; doi:10.3389/fmicb.2019.02372)

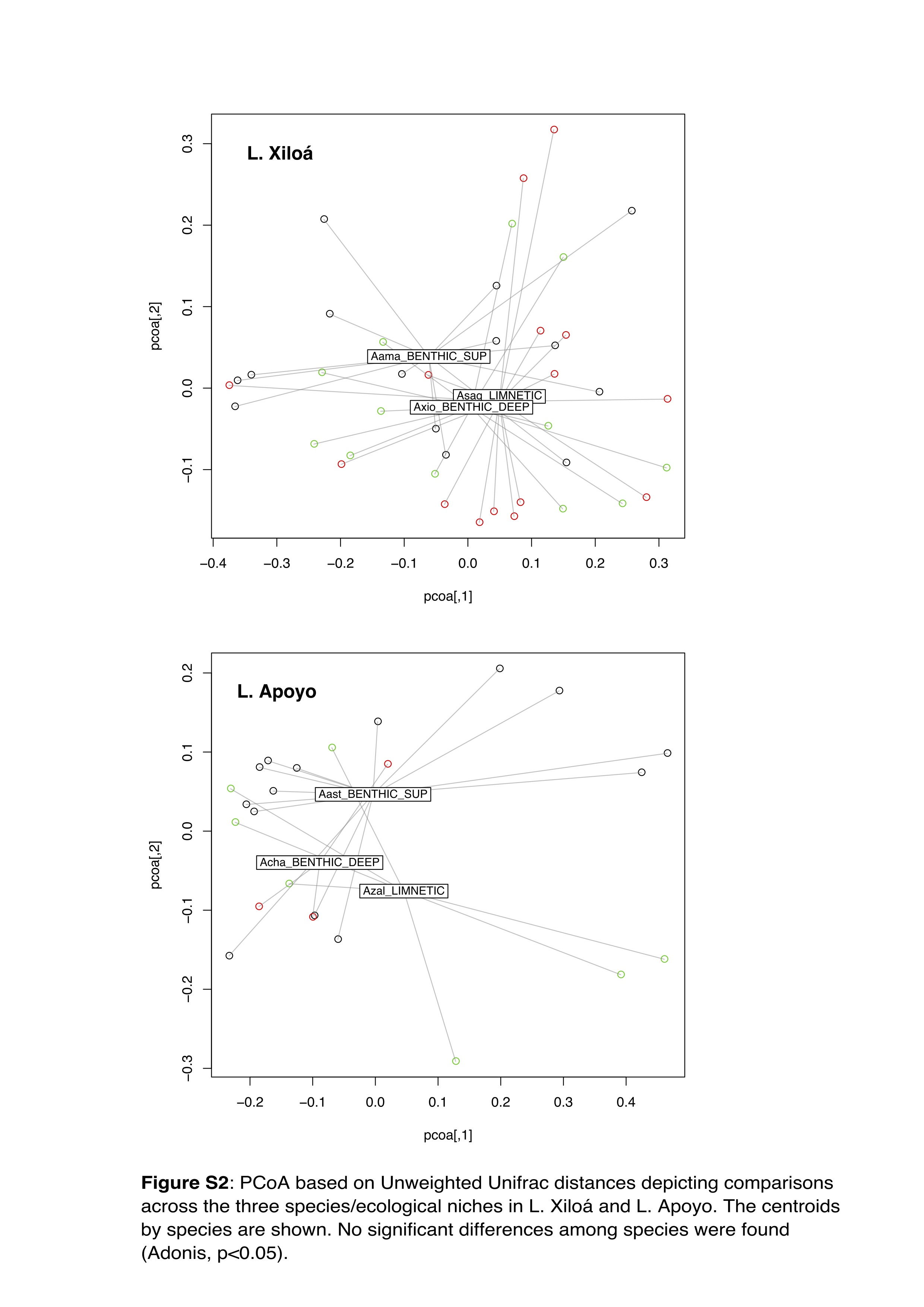

Supplement: FIGURE S2 — Principal Coordinates Analysis based on Unweighted Unifrac distances depicting comparisons across the three species/ecological niches in L. Xiloá and L. Apoyo. The centroids by species are shown. No significant differences among species were found (Adonis, p > 0.05). [file Image_2.JPEG]

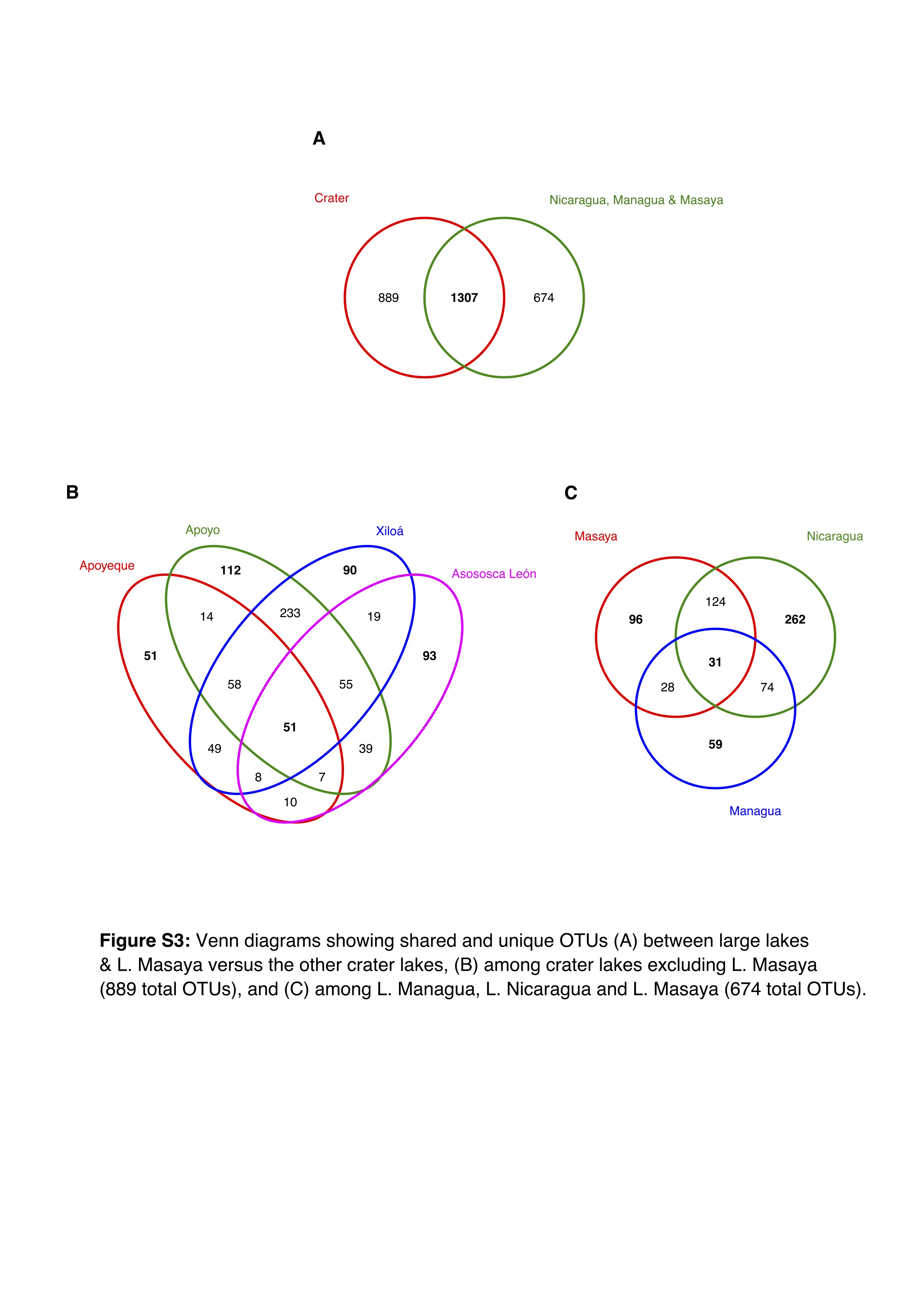

Supplement: FIGURE S3 — Venn diagrams showing shared and unique OTUs (A) between large lakes and L. Masaya versus the other crater lakes, (B) among crater lakes excluding L. Masaya (889 total OTUs), and (C) among L. Managua, L. Nicaragua and L. Masaya (674 total OTUs). [file Image_3.JPEG]
